# Adherence of HIV Self-Testing Among Men Who Have Sex With Men in China: Longitudinal Study

**DOI:** 10.2196/19627

**Published:** 2020-09-17

**Authors:** Xiangyu Yan, Hexuan Su, Bo Zhang, Yongjie Li, Lingling Zhang, Zhongwei Jia

**Affiliations:** 1 Department of Epidemiology and Biostatistics School of Public Health Peking University Beijing China; 2 National Institute on Drug Dependence Peking University Beijing China; 3 Medical Informatics Center Peking University Beijing China; 4 College of Nursing and Health Sciences University of Massachusetts Boston Boston, MA United States; 5 Center for Drug Abuse Control and Prevention National Institute of Health Data Science Peking University Beijing China; 6 Center for Intelligent Public Health Institute for Artificial Intelligence Peking University Beijing China

**Keywords:** HIV self-testing, adherence, men who have sex with men, HIV infection, condom use, mobile app

## Abstract

**Background:**

The World Health Organization recommended HIV self-testing (HIVST) for individuals practicing unsafe sexual behaviors; however, the adherence to HIV testing has not been reported.

**Objective:**

In this study, we attempted to determine the adherence to HIVST among men who have sex with men (MSM), as well as the impact factors and potential effects of their adherence.

**Methods:**

We conducted a longitudinal study among MSM in Harbin, Heilongjiang province, China from July 1, 2017 to June 30, 2018. A mobile app system was used to provide the “Mailing rapid test reagent kit” for the HIVST service. The proportion of those who adhered to HIV testing every 3 months was calculated. Logistic regression was used to explore the impact factors related to adherence to HIVST. Rates of HIV infection between MSM who adhered to HIVST and those who did not were compared using Cox proportional hazards regression. Changes of condom use behaviors between the two groups were also compared using the chi-square test.

**Results:**

A total of 1315 MSM who received the HIVST service through the app were included in the study. Overall, 10% of the MSM adhered to HIVST, and the proportion of adhering tests was only 34.9%. Adherence of HIVST was associated with marital status (adjusted odds ratio [OR]_unmarried vs married_ 2.31, 95% CI 1.13-4.71) and the number of HIV tests they received (adjusted OR_3 times vs 2 times or below_ 3.36, 95% CI 2.01-5.63; adjusted OR_4 times or above vs 2 times or below_ 7.30, 95% CI 4.67-11.42). Twenty HIV seroconversions were observed during 1-year follow up. The rate of HIV infection in the adherence group (17.10 per 100 person years, 95% CI 8.80-30.84) was significantly higher than that in the nonadherence group (4.80 per 100 person years, 95% CI 2.77-7.88; adjusted hazard ratio 3.33, 95% CI 1.35-8.20). Those who adhered to HIV testing were more likely to improve condom use behaviors, although the difference was not statistically significant.

**Conclusions:**

Regular HIV testing is necessary for early detection of HIV infection among MSM. Given the poor adherence, a new internet-based management paradigm for MSM is needed to raise their health awareness to optimize the implementation of HIVST.

## Introduction

Promoting HIV testing is an essential way to achieve the first 90% of the World Health Organization (WHO)’s “90-90-90” target (90% detection, 90% treatment, 90% viral suppression) [1]. However, in 2017, the gap to reach this first 90% remained large, with a worldwide detection rate of HIV infection of 75%, and about 30% of HIV-infected patients in China did not know their status [2,3]. The proportion was even larger among men who have sex with men (MSM); a systematic review indicated that about half of the MSM in China had not been tested for HIV [4]. In 2018, the theme of World AIDS Day was “Know Your Status,” which aimed to close the gap and expand HIV testing [5].

For key populations at high risk of HIV exposure, such as MSM, only paying attention to whether or not testing is performed is insufficient because it cannot reflect whether the frequency and regularity of HIV testing are appropriate [6,7]. Regular HIV testing is necessary for the early detection and treatment of HIV infection among MSM [8]. Considering the time window of 3-12 weeks of HIV detection along with the technical advances in HIV rapid test reagents, the Chinese Center for Disease Control and Prevention (CDC) recommended that sexually active MSM undergo HIV testing every 3 months, which is consistent with the recommendation of the US CDC and more frequent than the interval of 6-12 months recommended by the WHO in 2007 [8-10]. Despite the recommendation, it is important to examine the actual adherence to HIV testing among MSM to provide guidance for interventions. The Chinese government has made substantial effort in expanding HIV testing, with the number of HIV testing services provided by medical institutions increasing from 100 million in 2012 to 230 million in 2019 [11]. However, due to the anonymity in providing HIV testing services, it has not been possible to calculate the number of individuals who received HIV testing, let alone the testing frequency and adherence. Moreover, previous epidemiological studies rarely revealed the adherence to HIV testing but rather focused on the HIV testing rate or HIV testing uptake by examining whether MSM received HIV testing or not during the study period [12,13]. In addition, previous cross-sectional studies were not able to evaluate the real-world HIV testing adherence, as calculating this index requires follow-up observation, and participants in cohort studies and trials were influenced by the researchers’ reminders for receiving HIV testing and other services to avoid loss to follow up. In addition, questionnaire-based frequency of HIV testing might be a biased estimation, because the reporting bias was hard to measure.

Considering the inconvenience and perceived stigma raised from conventional facility-based HIV testing, HIV self-testing (HIVST) has been supported by 23 countries and was strongly recommended in the 2016 WHO guideline. HIVST is a process in which a person collects his/her own specimen (oral fluid or blood), performs a test, and interprets the result, often in a private setting, either alone or with a trusted person [14]. The “Thirteenth Five-year Plan” (2017-2022) for HIV prevention and control in China also supports HIVST [15,16]. Currently, with the increasing popularity of mobile devices and the rise in online sexual partners–seeking behaviors through mobile apps among MSM, a new method of HIVST named “Mailing rapid test reagent kit” has been conducted around the world. The implementation of this testing method, which combines internet technology with HIV testing and counseling services of community-based organizations, showed good acceptability and feasibility among MSM [17-19]. These findings shed light on the importance of the adherence to HIVST for development of HIVST policy and intervention methods. In the present study, we conducted a real-world longitudinal analysis of use of the “Mailing rapid test reagent kit” app for HIVST. We aimed to examine the adherence to HIV testing among MSM, as well as the impact factors and potential effects of their adherence.

## Methods

### Study Design and Setting

A longitudinal study was undertaken in Harbin, Heilongjiang province, China from July 1, 2017 to June 30, 2018. The participants were enrolled by the KangTong clinic, which is a community-based organization for MSM in Harbin. This site was chosen because it is the largest and most professional community-based organization in Harbin that could provide HIV testing services for MSM, and was considered to be the most acceptable site among MSM. In addition, the KangTong clinic uses the “Mailing rapid test reagent kit” as the main HIV testing service.

The inclusion criteria of participants were: (1) biologically male, (2) had anal sex with a man at least once in their life, (3) 15 years or older, (4) no difficulty in using the HIVST app, (5) willing to complete questionnaires of the study, and (6) willing to provide written informed consent. Participant recruitment was conducted continuously for a 1-year period. During recruitment, researchers and trained staff of the KangTong clinic publicized the HIVST app to MSM through electronic media (such as WeChat, QQ, and other geosocial networking apps) and offline venues (such as in clinics, parks, and bars) with the help of publicity posters and pictures. The HIVST app’s functions were introduced to participants by trained staff. The privacy policy was specifically explained to the participants, including: (1) the encrypted test result reports are only sent to the user through the app by an authorized person at the KangTong clinic; (2) the information collected will be kept confidential and anonymized; (3) if participants need consulting services related to the test results, the consulting service will be implemented in a one-to-one manner by trained staff of the community-based organization, and the process of consulting will also protect participants’ privacy. At the beginning of the registration, an electronic informed consent form was provided via the app. After completing registration and becoming familiar with HIVST functions, the community-based organization’s staff routinely informed participants of the benefits of HIV testing and encouraged them to receive the HIVST service through the app at any time as needed; however, no specific interventions on the frequency of using the HIVST service were implemented. Thus, we observed the frequency of using HIVST services based on individual behavior so as to best reflect adherence in a real-world situation.

Based on the frequency and interval between two adjacent HIVSTs, participants who met the following criteria were defined as the adherence group: (1) no interval between two adjacent HIV tests exceeded 100 days (including 100 days) and (2) the period between the date of the last HIV test and endpoint of this study (June 30, 2018) was less than or equal to 100 days. Participants who did not meet at least one of the above criteria were defined as the nonadherence group. However, for those with HIV seroconversion, if the last HIV test result was positive, and no further tests were required once an HIV infection was confirmed, the second criterion of adherence/nonadherence grouping was no longer considered. The threshold of 100 days was set according to the 3-month HIV testing interval recommended by the Chinese CDC, with consideration of weekends and holidays when the mail might be delayed. The primary outcome was adherence to HIV testing among MSM, and we also explored the impact factors of HIV testing adherence. The secondary outcomes were the rate of HIV infection and changes of condom use behaviors between the adherence and nonadherence groups.

Ethics approval for this study was obtained from the Peking University Institutional Review Board (IRB00001052-16016).

### HIVST Procedures

**Figure 1:**
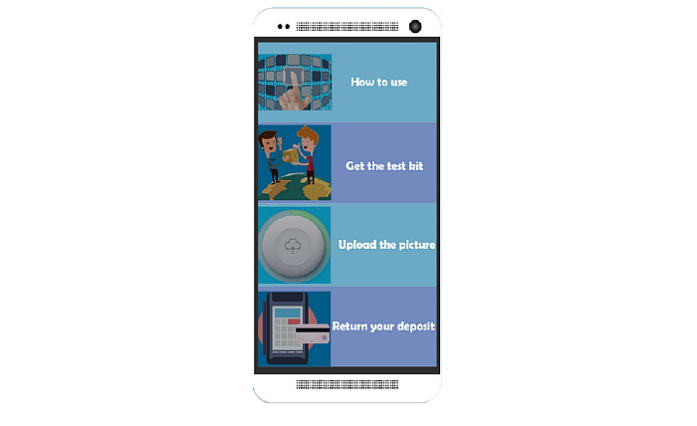
Interface of the “Mailing rapid test reagent kit” app.

**Figure 2:**
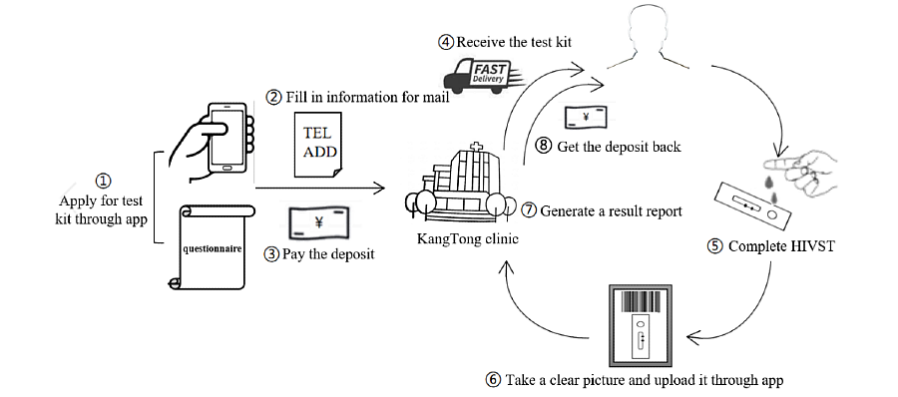
Flow chart of the “Mailing rapid test reagent kit” HIV self-testing (HIVST) service.

A new method of delivering the HIVST service “Mailing rapid test reagent kit” was supported by the KangTong clinic and provided to MSM through a mobile app. The procedure for using the app for HIVST was as follows: (1) click the “Get the test kit” button, and fill in a questionnaire related to sociodemographic characteristics, sexual behaviors, and condom use information; (2) fill in the phone number and address information used to mail the test kit; (3) pay a deposit (¥100, approximately US $14.22) through the app; (4) KangTong clinic sends a package including the HIV rapid test reagent kit (Alere Determine@) and two condoms to the user (the kit was provided free of charge, but an express shipping fee of ¥10 [approximately US $1.42] was required); (5) complete the HIVST using the test kit (a “How to use” button is available on the app including a tutorial video); (6) take a clear picture of the test kit and upload it to the KangTong clinic as required through the app interface (using the “Upload the picture” button); (7) the KangTong clinic generates an HIV testing report for the user in the app system showing the result (“Negative” or “Positive”) and date of this HIVST; (8) after receiving the report, the user can click the “Return your deposit” button, and get their deposit back (see Figure 1 and Figure 2). In addition, if a user’s test result was HIV positive, the staff of KangTong clinic would contact the user by phone initially. After checking basic information of the user to avoid contacting the wrong person, the staff would explain the meaning of the test result to the user and persuade him to come to the local CDC to confirm the HIV status accompanied by a staff member of the clinic. If the result was confirmed to be HIV-positive, the community-based organization’s staff would provide the psychological counseling service and help the user contact the local CDC for further treatment. The participant was informed of the availability of these follow-up services by community-based organization staff at the time of recruitment.

To protect user privacy, the encrypted test results were only sent to the user through the app by an authorized person at the KangTong clinic. The user could view the report after logging into the app. The user’s phone number and address were only used to mail the test kit and are kept confidential.

### Statistical Analysis

We calculated the proportion of MSM who adhered to HIV testing, and then compared characteristics between the adherence and nonadherence groups using the chi-square test. We used multiple imputations to deal with missing data in the variables of age, marital status, and age of first anal sex with a man because there was a less than 10% missing rate for these three variables. Univariate and multivariate logistic regression analyses were used to explore the impact factors of HIV testing adherence. For sensitivity analysis, different multivariate logistic regression models were constructed by adjusting using different variables to verify the stability of impact factors, and different multivariate logistic regression models were also constructed using original data with missing values. As another method to evaluate participants’ adherence to HIV testing, we also calculated the proportion of adhering tests. If the interval between a given HIV test and the previous test was shorter than 100 days (including 100 days), the given HIV test was defined as an “adhering test.” The proportion of adhering tests was calculated as the total number of adhering tests among participants divided by the total number of tests, excluding their first HIV tests.

Participants with at least two HIV test results were also included in the analysis to calculate the rates of HIV infection in the two groups. Because the enrollment time of each user was different, the follow-up period for each user was calculated individually. The date of HIV seroconversion was defined as the midway point between the last negative test date and the first positive test date. Univariate and multivariate Cox proportional hazards regression analyses were used to estimate the hazard ratio, reported with 95% CIs and *P* values, to compare the ability to detect HIV seroconversions according to adherence to HIV testing. The multivariate Cox proportional hazards regression model was adjusted for age, condom use behavior, and other sexually transmitted diseases (STDs).

The condom use behavior data were analyzed as a categorical variable based on the questionnaire response about condom use: “Never,” “Ever,” “Consistent,” and “Uncertain.” We considered the consistent use of condoms as a protective behavior. We divided the change of condom use behaviors into three categories: “Better,” “Worse,” and “Other.” “Better” means that the users changed their behavior to a more protective category (such as changing from “Never” to “Ever,” “Never” to “Consistent,” or “Ever” to “Consistent”), or maintained as “Consistent” (including individuals whose baseline information of condom use was uncertain, but consistent use of condom was reported in the follow up). “Worse” means that the users changed the behavior to a more unsafe category (such as changing from “Ever” to “Never,” “Consistent” to “Ever,” or “Consistent” to “Never”). “Other” means that the users’ condom use behaviors were maintained in the less protective categories (“Never” and “Ever”) and other conditions with uncertain condom use behavior. Participants with at least two HIV test results were included in the analysis to reflect the change of condom use behaviors. We compared the changes of condom use behaviors between the adherence and nonadherence groups using the chi-square test.

A two-sided *P* value of .05 or less was regarded as significant. The data were verified and statistical analyses were performed with SAS version 9.4 (SAS Institute, Inc., Cary, NC, USA) and SPSS version 21.0 (IBM Corp).

## Results

**Figure 3:**
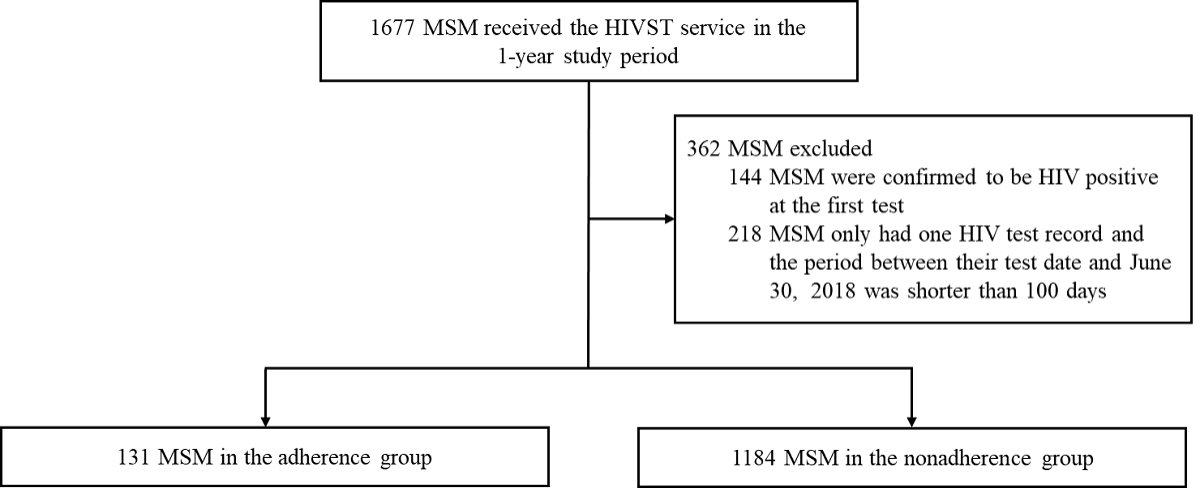
Study profile flowchart. HIVST: HIV self-testing: MSM: men who have sex with men.

From July 1, 2017 to June 30, 2018, a total of 2702 MSM were recorded as registered users of the app. Among them, 1677 (62.07%) MSM received the HIVST service and uploaded the test results. Data of 1315 app users were included in the final analysis and divided into the adherence group (n=131, 9.96%) and nonadherence group (n=1184, 90.04%) based on the intervals of their HIVSTs. Those who were confirmed to be HIV-positive at the first test (n=144) were excluded, and 218 MSM who only had one HIV test record and the period between their test date and endpoint of this study was shorter than 100 days were also excluded, because we could not confirm whether or not they would adhere to HIV testing (see Figure 3). A total of 2634 HIVSTs were received by the 1315 MSM. Among them, 920 HIVSTs (34.93%) were determined to be adhering tests.

As shown in Table 1, the majority of the 1315 MSM were unmarried and had attained higher education of university/college or above. With respect to sexual behaviors, although the majority of the participants reported first having anal sex with a man between 19 and 25 years of age, nearly a third of the participants reported their first such encounter when they were underage (≤18 years). Most of the MSM had always used the internet and some apps to seek sexual partners instead of traditional offline venues. Nearly 10% of the participants reported having had 3 or more casual man sex partners (9.1%) in the past 6 months, and approximately 15% also reported having had sex with women. With respect to drug use behaviors, approximately a quarter of the MSM used drugs (including methamphetamine, Rush Poppers, and other hallucinogens) to enhance sex. Approximately 10% reported a diagnosis of syphilis and other STDs.

**Table 1:** Basic characteristics of participants.

Characteristic				Total (N=1315), n (%)		Adherence to HIV testing (n=131), n (%)		Nonadherence to HIV testing (n=1184), n (%)					χ^2^	df		*P* value	
**Demographic and social characteristics**																	
	**Age (years)**										3.451			2		.18	
		≤25			362 (27.53)		33 (25.2)			329 (27.79)							
		26-40			525 (39.92)		46 (35.1)			479 (40.46)							
		≥41			428 (32.55)		52 (39.7)			376 (31.76)							
	**Marital status**										3.209			2		.20	
		Married			222 (16.88)		15 (11.5)			207 (17.48)							
		Unmarried			1038 (78.94)		111 (84.7)			927 (78.29)							
		Divorced or widowed			55 (4.18)		5 (3.8)			50 (4.22)							
	**Education**										1.816			2		.40	
		Junior high school or below			188 (14.30)		16 (12.2)			172 (14.53)							
		High school			284 (21.60)		24 (18.3)			260 (21.96)							
		University/college or above			843 (64.11)		91 (69.5)			752 (63.51)							
**Sexual behaviors**																	
	**Age of first anal sex with a man (years)**										1.309			3		.73	
			≤18		373 (28.37)		32 (24.4)			341 (28.80)							
			19-25		689 (52.40)		71 (54.2)			618 (52.20)							
			26-40		203 (15.44)		23 (17.6)			180 (15.20)							
			≥41		50 (3.80)		5 (3.8)			45 (3.80)							
	**Sex role**										2.856			2		.24	
			Insertive only		475 (36.12)		56 (42.7)			419 (35.39)							
			Receptive only		322 (24.49)		30 (22.9)			292 (24.66)							
			Both		518 (39.39)		45 (34.4)			473 (39.95)							
	**Place for seeking sexual partners**										1.751			1		.19	
			Offline venues (eg, bars, parks)		269 (20.46)		21 (16.0)			248 (20.95)							
			Internet/software/app		1046 (79.54)		110 (84.0)			936 (79.05)							
	**Number of regular man sexual partners in the past 6 months**										5.499			3		.14	
			0		543 (41.29)		51 (38.9)			492 (41.55)							
			1		514 (39.09)		50 (38.2)			464 (39.19)							
			2		174 (13.23)		25 (19.1)			149 (12.58)							
			3 or above		84 (6.39)		5 (3.8)			79 (6.67)							
	**Number of casual man sexual partners (with no money transaction) in the past 6 months**										3.397			3		.33	
			0		735 (55.89)		68 (51.9)			667 (56.33)							
			1		295 (22.43)		28 (21.4)			267 (22.55)							
			2		165 (12.55)		23 (17.5)			142 (11.99)							
			3 or above		120 (9.13)		12 (9.2)			108 (9.12)							
	**Sex with male sex workers in the past 6 months**										0.891			1		.35	
			No		1256 (95.51)		123 (93.9)			1133 (95.69)							
			Yes		59 (4.49)		8 (6.1)			51 (4.31)							
	**Sex with women in the past 6 months**										1.957			1		.16	
			No		1109 (84.33)		116 (88.5)			993 (83.87)							
			Yes		206 (15.67)		15 (11.5)			191 (16.13)							
	**Drug use for enhancing sex in the past 6 months**										2.313			1		.13	
			No		1004 (76.35)		93 (71.0)			911 (76.94)							
			Yes		311 (23.65)		38 (29.0)			273 (23.06)							
**HIV and STDs^a^**																	
	**HIV seroconversion**										20.429			1		<.001	
			No		1295 (98.48)		123 (93.9)			1172 (98.99)							
			Yes		20 (1.52)		8 (6.1)			12 (1.01)							
	**Other STDs**										0.028			1		.87	
			No		1180 (89.73)		117 (89.3)			1063 (89.78)							
			Yes		135 (10.27)		14 (10.7)			121 (10.22)							
	**Number of HIVSTs^b^**										103.031			2		<.001	
			2 times or below		992 (75.44)		55 (42.0)			937 (79.14)							
			3 times		155 (11.79)		26 (19.8)			129 (10.90)							
			4 times or above		168 (12.78)		50 (38.2)			118 (9.97)							

^a^STD: sexually transmitted disease.

^b^HIVST: HIV self-testing.

The baseline characteristics of MSM from the adherence and nonadherence groups were similar, with no significant differences observed in terms of sociodemographic characteristics and sexual behaviors. However, some notable differences were found between the groups, including a larger proportion of HIV seroconversions in the adherence group than in the nonadherence group, and MSM in the adherence group tended to have more HIVSTs compared with those in the nonadherence group (Table 1).

Regression analysis showed that MSM who were unmarried were more likely than those who were married to women to adhere to HIVST. In comparison with MSM who had 2 or less HIVSTs, those who had 3 or more tests were more likely to adhere to HIVST (Table 2). No other significant factors contributing to HIVST adherence were found among the sexual behaviors and STD variables. The results remained stable in sensitivity analysis when variables were adjusted differently in different multivariate logistic regression models (see Multimedia Appendix 1).

At 1-year follow-up, a total of 20 seroconversions were found, including 8 in the adherence group and 12 in the nonadherence group. The rate of HIV infection in the adherence group was significantly higher than that in the nonadherence group (Table 3).

With respect to condom use behaviors, almost half of the MSM used condoms consistently in their recent anal sex with men at baseline at the time of their first HIV tests, and only 1.5% never used a condom. The differences in condom use behaviors between the two groups were not significant at baseline (χ^2^_3_=3.096, *P*=.38; Table 4). However, most of the MSM had changed the behaviors to a better level or maintained the consistent use of condoms by the 1-year follow-up. In addition, the proportion of MSM whose behaviors became “Better” was higher in the adherence group than that in the nonadherence group when comparing the behaviors at their second HIV tests to the baseline, and the result was similar when comparing the behaviors at their fourth HIV tests to the baseline; although none of the differences between the two groups was statistically significant, the implications are meaningful (Table 5, Multimedia Appendix 2). The proportions of behaviors becoming “Better” in the two groups were similar when comparing the third HIV testing to the baseline (see Table 5, Multimedia Appendix 2).

**Table 2:** Impact factors of HIV self-testing (HIVST) adherence.

Characteristic			Proportion, % (n/N)^a^	OR^b^ (95%CI)	*P* value	AOR^c^ (95%CI)	*P* value
**Demographic and social characteristics**							
	**Age (years)**						
		≤25	9.1 (33/362)	1.00	N/A^d^	1.00	N/A
		26-40	8.8 (46/525)	0.99 (0.61-1.59)	.96	0.99 (0.58-1.70)	.98
		≥41	12.1 (52/428)	1.44 (0.90-2.30)	.13	1.66 (0.96-2.90)	.07
	**Marital status**						
		Married	6.8 (15/222)	1.00	N/A	1.00	N/A
		Unmarried	10.7 (111/1038)	1.65(0.94-2.88)	.08	2.31 (1.13-4.71)	.02
		Divorced or widowed	9.1 (5/55)	1.36 (0.47-3.92)	.57	1.27 (0.41-3.94)	.68
	**Education**						
		Junior high school or below	8.5 (16/188)	1.00	N/A	1.00	N/A
		High school	8.5 (24/284)	0.99 (0.51-1.92)	.98	0.89 (0.44-1.80)	.74
		University/college or above	10.8 (91/843)	1.30 (0.75-2.27)	.35	0.96 (0.52-1.79)	.91
**Sexual behaviors**							
	**Age of first anal sex with a man (years)**						
		≤18	8.6 (32/373)	1.00	N/A	1.00	N/A
		19-25	10.3 (71/689)	1.24 (0.80-1.93)	.33	1.23 (0.76-1.98)	.41
		26-40	11.3 (23/203)	1.35 (0.77-2.38)	.30	1.69 (0.86-3.31)	.13
		≥41	10.0 (5/50)	1.05 (0.36-3.08)	.93	1.55 (0.46-5.20)	.48
	**Sex role**						
		Insertive only	11.8 (56/475)	1.00	N/A	1.00	N/A
		Receptive only	9.3 (30/322)	0.77 (0.48-1.23)	.27	0.88 (0.52-1.48)	.63
		Both	8.7 (45/518)	0.71 (0.47-1.08)	.11	0.79 (0.51-1.24)	.31
	**Place for seeking sexual partners**						
		Offline venues (eg, bars, parks)	7.8 (21/269)	1.00	N/A	1.00	N/A
		Internet/software/app	10.5 (110/1046)	1.39 (0.85-2.26)	.19	1.50 (0.89-2.54)	.13
	**Number of regular man sexual partners in the past 6 months**						
		0	9.4 (51/543)	1.00	N/A	1.00	N/A
		1	9.7 (50/514)	1.04 (0.69-1.57)	.85	0.87 (0.54-1.40)	.57
		2	14.4 (25/174)	1.62 (0.97-2.70)	.07	1.35 (0.72-2.54)	.36
		3 or above	6.0 (5/84)	0.61 (0.24-1.58)	.31	0.50 (0.18-1.39)	.19
	**Number of casual man sexual partners (with no money transaction) in the past 6 months**						
		0	9.3 (68/735)	1.00	N/A	1.00	N/A
		1	9.5 (28/295)	1.03 (0.65-1.63)	.91	0.92 (0.54-1.55)	.75
		2	13.9 (23/165)	1.59 (0.96-2.64)	.07	1.12 (0.62-2.05)	.70
		3 or above	10.0 (12/120)	1.09 (0.57-2.08)	.79	1.12 (0.53-2.33)	.77
	**Had sex with male sex workers in the past 6 months**						
		No	9.8 (123/1256)	1.00	N/A	1.00	N/A
		Yes	13.6 (8/59)	1.45 (0.67-3.11)	.35	1.56 (0.66-3.71)	.31
	**Had sex with women in the past 6 months**						
		No	10.5 (116/1109)	1.00	N/A	1.00	N/A
		Yes	7.3 (15/206)	0.67 (0.38-1.18)	.16	0.84 (0.45-1.58)	.59
	**Drug use for enhancing sex in the past 6 months**						
		No	9.3 (93/1004)	1.00	N/A	1.00	N/A
		Yes	12.2 (38/311)	1.36 (0.91-2.04)	.13	1.20 (0.75-1.92)	.44
**STDs^e^**							
	**Have other STDs**						
		No	9.9 (117/1180)	1.00	N/A	1.00	N/A
		Yes	10.4 (14/135)	1.05 (0.58-1.89)	.87	1.03 (0.54-1.94)	.94
	**Number of HIVSTs**						
		2 times or below	5.5 (55/992)	1.00	N/A	1.00	N/A
		3 times	16.8 (26/155)	3.43 (2.08-5.67)	<.001	3.36 (2.01-5.63)	<.001
		4 times or above	29.8 (50/168)	7.22 (4.70-11.08)	<.001	7.30 (4.67-11.42)	<.001

^a^Proportion=number of participants in adherence group/total number of participants in the category.

^b^OR: odds ratio.

^c^AOR: adjusted odds ratio; all characteristics were adjusted in the multivariate logistic model.

^d^N/A: not applicable.

^e^STD: sexually transmitted disease.

**Table 3:** Comparison of HIV infection rates of the adherence and nonadherence groups.

Group	Seroconversions	Person years	Rate^a^ (95% CI)	HR^b^ (95%CI)	*P* value	AHR^c^ (95%CI)	*P* value
Total^d^	20	296.69	6.74 (4.38-10.00)	N/A^e^	N/A	N/A	N/A
Nonadherence to HIV testing	12	249.91	4.80 (2.77-7.88)	1.00	N/A	1.00	N/A
Adherence to HIV testing	8	46.77	17.10 (8.80-30.84)	3.48 (1.42-8.54)	.006	3.33 (1.35-8.20)	.009

^a^Rate is calculated as the number of seroconversions per 100 person years.

^b^HR: hazard ratio.

^c^AHR: adjusted hazard ratio; multivariate Cox proportional hazards model adjusted for age, condom use behaviors, and other sexually transmitted diseases.

^d^A total of 680 men who have sex with men with 2 or more HIV tests were included in this analysis.

^e^N/A: not applicable.

**Table 4:** Comparison of baseline condom use behaviors in the adherence and nonadherence groups.

Condom usage	Total (N=680), n (%)	Adherence to HIV testing (n=131), n (%)	Nonadherence to HIV testing (n=549), n (%)
Never	10 (1.5)	4 (3.1)	6 (1.1)
Ever	162 (23.8)	30 (22.9)	132 (24.0)
Consistent	305 (44.9)	56 (42.7)	249 (45.4)
Uncertain	203 (29.8)	41 (31.3)	162 (29.5)

**Table 5:** Comparison of condom use behavior changes in the adherence and nonadherence groups.

Testing interval		Adherence to HIV testing, n (%)		Nonadherence to HIV testing, n (%)		χ^2^ (df=2)		*P* value
**Second test vs first test (n=680)**						1.202		.55
	Better		65 (49.6)		244 (44.5)			
	Worse		15 (11.5)		65 (11.8)			
	Other		51 (38.9)		240 (43.7)			
**Third test vs first test (n=323)**						0.539		.76
	Better		31 (40.8)		103 (41.7)			
	Worse		12 (15.8)		31 (12.6)			
	Other		33 (43.4)		113 (45.7)			
**Fourth test vs first test (n=168)**						1.109		.57
	Better		29 (58.0)		58 (49.1)			
	Worse		4 (8.0)		12 (10.2)			
	Other		17 (34.0)		48 (40.7)			

## Discussion

### Principal Findings

In this study, we estimated the HIV adherence among MSM using first-hand data recorded by the app of HIVST, demonstrating that about 90% of MSM do not adhere to HIV testing as recommended by the Chinese CDC without targeted intervention. The proportion was much higher than that lost to follow up (23%-54%) reported by cohort studies and trials that provided HIV testing services [20-22]. In addition, the proportion of adhering tests was only 34.9%. These results indicate the need to focus on the poor HIV testing adherence among MSM.

The negative attitude of MSM toward regular HIVST might be related to certain social and cultural factors. First, MSM may not have sufficient awareness of the need for regular HIV testing because of the lack of related publicity and education [23,24]. The education of HIV testing for MSM has mainly focused on the action to persuade them to be tested and to know their HIV status; however, only focusing on whether they have had HIV test results in their lifetime and the number of tests they received is inadequate, as promoting regular HIV testing is also important [24,25]. Unfortunately, the mobilization for “regular testing” of sexually active people is inadequate all over the world [24-28]. Thus, greater awareness and habit of adhering to HIV testing need to be cultivated. In this study, we found that MSM who received more HIV testing had better HIV testing adherence (adjusted odds ratio_3 times_ 3.36, 95% CI 2.01-5.63; adjusted odds ratio_4 times or above_ 7.30, 95% CI 4.67-11.42), which showed that improving HIV testing adherence is possible once the habit of regular testing is cultivated, and targeted interventions are needed to focus on MSM who are in the habit-formation period.

Second, MSM in China still face prejudice, discrimination, and stigma associated with their sexual orientation and same-sex behavior [27]. This phenomenon is related to the social and cultural environment of China, although it has improved in recent years, which might still hinder MSMs’ adherence to HIV testing. Our study showed that MSM who were unmarried were more likely to adhere to HIV testing than those married to women (adjusted odds ratio 2.31, 95% CI 1.13-4.71). The majority of MSM who were married to women did not want to disclose their male sex behaviors and risk of HIV infection to their families. The stigma and psychological distress from privacy disclosure might prevent them from receiving HIV testing regularly [29].

Third, MSM, especially those with persistent high-risk behaviors, are prone to denial of the risk of HIV infection; thus, frequent testing may introduce greater psychological stress [27]. In addition, several studies have shown that economic factors and testing costs are the main factors restricting the use of testing services [23,27,30]. However, in our study, the testing reagent kits were provided free of charge, and only a small express fee was required, which could effectively avoid the influence of economic and cost factors on testing intentions.

We found that adhering to HIV testing regularly had two potential beneficial effects. First, it could promote case identification of HIV-infected MSM. The rate of HIV infection in the adherence group was 3.3 times higher than that in the nonadherence group (95% CI 1.35-8.20). Given the similar characteristics of sexual behaviors and STDs between the two groups, this effect of case identification was mainly attributable to the timely and regular testing, which could contribute to realization of the first “90%” in the WHO’s 90-90-90 target and improve the effectiveness of HIV testing services. Second, adhering to HIV testing might have a potential positive impact on condom use behaviors. There was a tendency for those adhering to HIV testing to be more likely to improve their condom use behavior than those who did not adhere to testing (5% better at their second HIVST, 9% better at their fourth HIVST). Although the difference was not statistically significant, this beneficial effect might be meaningful because the goal of high-risk behavior reduction among MSM was only 10% in the “Thirteenth Five-year Plan” (2017-2022) for HIV prevention and control in China, and adhering to HIVST within the 1-year period of this study could achieve a 5% reduction in high-risk sexual behavior [9]. Tang and colleagues [31] showed that HIVST was associated with subsequent consistent condom use, which also supports our finding.

Therefore, it is necessary to improve the low level of HIV testing adherence. An internet-based HIVST service such as “Mailing rapid test reagent kit” could be a feasible method to promote the adherence and regularity of HIV testing. The main benefits of such a service include dependable privacy protection, cost-effectiveness, and acceptability [32-34]. In this study, 62.07% (1677/2702) of the registered users of the app received the HIVST service and uploaded the test results, which is much higher than the proportion of 21.4% reported by De Boni et al [35]. In addition, approximately a quarter of registered app users (680/2702) received more than one test. Moreover, the internet-based method could be used as a feasible and utilizable tool to solve the following challenges brought by use of the internet in HIV/AIDS control for MSM and to promote case identification. On the one hand, the internet has gradually become the main source of seeking sexual partners for MSM [36]. Indeed, in our study, about 80% of MSM sought sexual partners using internet software or apps, which are more convenient but also bring about more challenges for HIV transmission [37]. On the other hand, more than a quarter of the MSM in our study (28.4%) first reported having anal sex when they were underage. There is a trend that internet users tend to be younger in China [38]. Quayle and Newman [39] indicated that some images and messages with sexual inducement on the internet offered sexual motivation and interest to users, especially for minors. Therefore, it is necessary to pay attention to the internet-based HIV prevention and testing services for minors. In addition, 23.7% of the MSM in this study reported using drugs for enhancing sex, and recent studies have shown that the internet is facilitating drug trafficking [40]. Drug use could result in the loss of self-control and is often accompanied by high-risk sexual behaviors that increase the risk of HIV transmission [41].

Given that the HIV testing adherence was only 10% in this study, we should consider whether the HIVST service provided needs to be improved by considering aspects of empowerment and feasibility. Although the HIVST package is free and convenient to use, HIVST users may feel greater distress, anxiety, and less social support when testing at home, especially for the first test. However, with the increase of the number of tests, this psychological pressure will be gradually diluted [42], which further highlights the importance of regular testing. Enabling MSM to perform self-testing at home may increase their sense of empowerment or self-assurance by taking charge of their own health [43], and then eventually form a benign positive feedback mechanism. Therefore, targeted improvements of interventions along with internet-based HIVST are essential. More online health education needs to be provided to promote MSMs’ awareness of not only HIV testing but also the importance of adhering to HIV testing as far as possible to reduce the threshold of inner stigmas. In addition, improving the participants’ empowerment might be a gradual process. To cultivate their habit of adhering to HIV testing, a module can be added to internet-based HIVST tools to automatically remind MSM to receive HIV testing regularly based on their previous test records.

### Limitations

Our study has several limitations. First, the 1-year observational period was relatively short, and only 20 seroconversions were found during this time, which might lead to a biased estimation of the rate of HIV infection. Second, our study was only conducted at a single site in an urban area of Harbin and overlooked the HIV testing adherence in rural areas, which will need further investigations. Finally, some people might have purchased an HIV testing kit on their own or received HIV testing in other cities, which might have resulted in underestimating the HIV testing adherence in our study. Nevertheless, our study clearly showed the adherence to the “Mailing rapid test reagent kit” HIVST service in Harbin.

To our knowledge, this is the first study that aimed to focus on adherence to HIV testing in a real-world situation. The characteristics of MSM who adhered to HIV testing regularly in this study could serve as an important external control for future studies and provide good evidence for guideline development in HIV testing services. In future studies, researchers should recruit a larger number of MSM participants at multiple centers with a much longer follow-up period to further estimate HIV testing adherence among MSM.

### Conclusions

In this longitudinal study, only 10% of participants adhered to HIVST using the app of “Mailing rapid test reagent kit.” Regular HIV testing was shown to be necessary for the early detection of HIV infection and might potentially promote condom use behaviors to some extent. Our study has major practical implications for public health decisions and policymakers. In particular, more attention should be paid to encourage regular HIV testing rather than only recommending HIV testing among sexually active subpopulation at high risk of HIV exposure. HIV testing adherence could become an indicator recommended in guidelines to measure the implementation quality of HIV testing. More feasible and affordable public health management paradigms combined with internet-based methods could be implemented to promote the adherence and regularity of HIV testing.

## Appendix

Multimedia Appendix 1 Different models to explore impact factors of HIV self-testing (HIVST) adherence.

Multimedia Appendix 2 Follow-up condom use behaviors changes compared with the baseline. AHT: adherence to HIV testing; MSM: men who have sex with men.

## Abbreviations

CDC: Center for Disease Control and Prevention
HIVST: HIV self-testing
MSM: men who have sex with men
STD: sexually transmitted disease
WHO: World Health Organization
