# Supplementary material for: Adherence of HIV Self-Testing Among Men Who Have Sex With Men in China: Longitudinal Study
Source: J Med Internet Res. 2020 Sep 17;22(9):e19627. doi: 10.2196/19627 (PMC7530689; doi:10.2196/19627)
Supplement: Multimedia Appendix 1 [file jmir_v22i9e19627_app1.docx]

**Different models to explore impact factors of HIVST adherence (multiple imputations)**

|  |  | **Model 1*** | |  | **Model 2**** | |  | **Model 3***** | |
| --- | --- | --- | --- | --- | --- | --- | --- | --- | --- |
|  |  | **AOR (95%CI)** | **p value** |  | **AOR (95%CI)** | **p value** |  | **AOR (95%CI)** | **p value** |
| **Demographic and social characteristics** | | |  |  |  |  |  |  |  |
| Age (years) | |  |  |  |  |  |  |  |  |
|  | ≤25 | 1.00 | - |  | 1.00 | - |  | 1.00 | - |
|  | 26-40 | 1.03(0.62-1.70) | .91 |  | 1.04(0.63-1.74) | .88 |  | 1.03(0.62-1.70) | .91 |
|  | ≥41 | 1.59(0.95-2.67) | .08 |  | 1.86(1.10-3.16) | .02 |  | 1.59(0.95-2.66) | .08 |
| Marital status | |  |  |  |  |  |  |  |  |
|  | Married | 1.00 | - |  | 1.00 | - |  | 1.00 | - |
|  | Unmarried | 2.07(1.06-4.07) | .03 |  | 2.13(1.13-4.03) | .02 |  | 2.07(1.06-4.07) | .03 |
|  | Divorced or widowed | 1.28(0.43-3.81) | .66 |  | 1.23(0.41-3.71) | .72 |  | 1.28(0.43-3.81) | .66 |
| Education | |  |  |  |  |  |  |  |  |
|  | Junior high school or below | 1.00 | - |  | 1.00 | - |  | 1.00 | - |
|  | High school | 0.87(0.44-1.72) | .70 |  | 0.91(0.45-1.81) | .78 |  | 0.87(0.44-1.72) | .70 |
|  | University/college or above | 1.13(0.63-2.04) | .68 |  | 0.98(0.54-1.79) | .94 |  | 1.13(0.63-2.04) | .68 |
| **Sexual behaviors** | |  |  |  |  |  |  |  |  |
| Age of first anal sex with man (years) | | |  |  |  |  |  |  |  |
|  | ≤18 | 1.00 | - |  | - | - |  | 1.00 | - |
|  | 19-25 | 1.24(0.78-1.97) | .35 |  | - | - |  | 1.24(0.79-1.97) | .35 |
|  | 26-40 | 1.76(0.93-3.35) | .08 |  | - | - |  | 1.77(0.93-3.35) | .08 |
|  | ≥41 | 1.54(0.47-5.06) | .47 |  | - | - |  | 1.55(0.47-5.08) | .47 |
| Sex role | |  |  |  |  |  |  |  |  |
|  | Insertive only | 1.00 | - |  | - | - |  | 1.00 | - |
|  | Receptive only | 0.75(0.46-1.22) | .24 |  | - | - |  | 0.75(0.46-1.22) | .24 |
|  | Both | 0.72(0.47-1.11) | .13 |  | - | - |  | 0.72(0.47-1.11) | .13 |
| Place for seeking sexual partners | | |  |  |  |  |  |  |  |
|  | Offline venues (bars, parks and so on) | 1.00 | - |  | - | - |  | 1.00 | - |
|  | Internet/software/app | 1.42(0.86-2.34) | .18 |  | - | - |  | 1.42(0.86-2.34) | .18 |
| Number of regular man sexual partners in the past six months | | | | | |  |  |  |  |
|  | 0 | 1.00 | - |  | - | - |  | 1.00 | - |
|  | 1 | 0.91(0.58-1.43) | .68 |  | - | - |  | 0.91(0.58-1.43) | .68 |
|  | 2 | 1.27(0.70-2.28) | .43 |  | - | - |  | 1.26(0.70-2.28) | .44 |
|  | 3 or above | 0.51(0.19-1.37) | .18 |  | - | - |  | 0.51(0.19-1.38) | .18 |
| Number of casual man sexual partners (with no money transaction) in the past six months | | | | | | |  |  |  |
|  | 0 | 1.00 | - |  | - | - |  | 1.00 | - |
|  | 1 | 0.93(0.56-1.54) | .79 |  | - | - |  | 0.93(0.56-1.54) | .78 |
|  | 2 | 1.37(0.78-2.42) | .27 |  | - | - |  | 1.37(0.78-2.42) | .27 |
|  | 3 or above | 1.03(0.51-2.06) | .95 |  | - | - |  | 1.02(0.51-2.05) | .95 |
| Have sex with male sex workers in the past six months | | | |  |  |  |  |  |  |
|  | No | 1.00 | - |  | - | - |  | 1.00 | - |
|  | Yes | 1.50(0.67-3.36) | .33 |  | - | - |  | 1.49(0.66-3.36) | .33 |
| Have sex with women in the past six months | | |  |  |  |  |  |  |  |
|  | No | 1.00 | - |  | - | - |  | 1.00 | - |
|  | Yes | 0.68(0.37-1.25) | .22 |  | - | - |  | 0.68(0.37-1.25) | .22 |
| Drug use for promoting sex in the past six months | | | |  |  |  |  |  |  |
|  | No | 1.00 | - |  | - | - |  | 1.00 | - |
|  | Yes | 1.30(0.83-2.01) | .25 |  | - | - |  | 1.29(0.83-2.01) | .25 |
| **STDs** | |  |  |  |  |  |  |  |  |
| Have other STDs | |  |  |  |  |  |  |  |  |
|  | No | - | - |  | 1.00 | - |  | 1.00 | - |
|  | Yes | - | - |  | 1.09(0.59-2.03) | .79 |  | 1.03(0.56-1.88) | .93 |
| **HIVST behaviors** | |  |  |  |  |  |  |  |  |
| Number of HIVSTs | |  |  |  |  |  |  |  |  |
|  | 2 times or below | - | - |  | 1.00 | - |  | - | - |
|  | 3 times | - | - |  | 3.57(2.15-5.93) | <.001 |  | - | - |
|  | 4 times or above | - | - |  | 7.27(4.70-11.25) | <.001 |  | - | - |

AOR: adjusted odds ratio; STD: sexually transmitted disease; HIVST: HIV self-testing.

*****Demographic and social characteristics, sexual behaviors were adjusted in the multivariate logistic model.

******Demographic and social characteristics, STDs, and HIVST behaviors were adjusted in the multivariate logistic model.

*******Demographic and social characteristics, sexual behaviors, and STDs were adjusted in the multivariate logistic model.

**Different models to explore impact factors of HIVST adherence (original data with missing data)**

|  |  | **Model 1*** | |  | **Model 2**** | |  | **Model 3***** | | **Model 4****** | |
| --- | --- | --- | --- | --- | --- | --- | --- | --- | --- | --- | --- |
|  |  | **AOR (95%CI)** | **p value** |  | **AOR (95%CI)** | **p value** |  | **AOR (95%CI)** | **p value** | **AOR (95%CI)** | **p value** |
| **Demographic and social characteristics** | | |  |  |  |  |  |  |  |  |  |
| Age (years) | |  |  |  |  |  |  |  |  |  |  |
|  | ≤25 | 1.00 | - |  | 1.00 | - |  | 1.00 | - | 1.00 | - |
|  | 26-40 | 1.05(0.63-1.74) | .86 |  | 1.08(0.65-1.80) | .76 |  | 1.04(0.63-1.74) | .87 | 1.02(0.59-1.75) | .94 |
|  | ≥41 | 1.61(0.95-2.70) | .08 |  | 1.87(1.10-3.18) | .02 |  | 1.60(0.95-2.70) | .08 | 1.64(0.95-2.86) | .08 |
|  | Missing data | 2.53(0.99-6.50) | .05 |  | 2.28(0.87-5.95) | .09 |  | 2.54(0.99-6.51) | .05 | 2.29(0.84-6.26) | .11 |
| Marital status | |  |  |  |  |  |  |  |  |  |  |
|  | Married | 1.00 | - |  | 1.00 | - |  | 1.00 | - | 1.00 | - |
|  | Unmarried | 2.05(1.04-4.03) | .04 |  | 2.03(1.08-3.82) | .03 |  | 2.06(1.05-4.04) | .04 | 2.27(1.12-4.61) | .02 |
|  | Divorced or widowed | 1.28(0.43-3.82) | .66 |  | 1.19(0.39-3.61) | .76 |  | 1.28(0.43-3.81) | .66 | 1.27(0.41-3.93) | .68 |
|  | Missing data | - | - |  | - | - |  | - | - |  |  |
| Education | |  |  |  |  |  |  |  |  |  |  |
|  | Junior high school or below | 1.00 | - |  | 1.00 | - |  | 1.00 | - | 1.00 | - |
|  | High school | 0.86(0.43-1.69) | .66 |  | 0.91(0.45-1.82) | .79 |  | 0.85(0.43-1.69) | .65 | 0.89(0.44-1.81) | .74 |
|  | University/college or above | 1.12(0.62-2.02) | .70 |  | 0.99(0.54-1.81) | .98 |  | 1.13(0.62-2.03) | .69 | 0.98(0.53-1.83) | .95 |
| **Sexual behaviors** | |  |  |  |  |  |  |  |  |  |  |
| Age of first anal sex with man (years) | | |  |  |  |  |  |  |  |  |  |
|  | ≤18 | 1.00 | - |  | - | - |  | 1.00 | - | 1.00 | - |
|  | 19-25 | 1.32(0.81-2.13) | .26 |  | - | - |  | 1.32(0.81-2.14) | .26 | 1.29(0.78-2.13) | .32 |
|  | 26-40 | 1.92(0.99-3.74) | .05 |  | - | - |  | 1.93(0.99-3.75) | .05 | 1.85(0.92-3.71) | .08 |
|  | ≥41 | 1.65(0.49-5.54) | .41 |  | - | - |  | 1.66(0.50-5.58) | .41 | 1.80(0.52-6.19) | .35 |
|  | Missing data | 1.83(0.81-4.11) | .14 |  | - | - |  | 1.84(0.81-4.13) | .14 | 1.31(0.56-3.07) | .53 |
| Sex role | |  |  |  |  |  |  |  |  |  |  |
|  | Insertive only | 1.00 | - |  | - | - |  | 1.00 | - | 1.00 | - |
|  | Receptive only | 0.75(0.46-1.22) | .25 |  | - | - |  | 0.75(0.46-1.22) | .25 | 0.88(0.52-1.48) | .63 |
|  | Both | 0.74(0.48-1.13) | .16 |  | - | - |  | 0.74(0.48-1.13) | .16 | 0.80(0.51-1.25) | .33 |
| Place for seeking sexual partners | | |  |  |  |  |  |  |  |  |  |
|  | Offline venues (bars, parks and so on) | 1.00 | - |  | - | - |  | 1.00 | - | 1.00 | - |
|  | Internet/software/app | 1.50(0.90-2.50) | .12 |  | - | - |  | 1.50(0.90-2.50) | .12 | 1.54(0.90-2.63) | .11 |
| Number of regular man sexual partners in the past six months | | | | | |  |  |  |  |  |  |
|  | 0 | 1.00 | - |  | - | - |  | 1.00 | - | 1.00 | - |
|  | 1 | 0.93(0.59-1.47) | .76 |  | - | - |  | 0.93(0.59-1.47) | .76 | 0.88(0.55-1.42) | .61 |
|  | 2 | 1.32(0.73-2.39) | .36 |  | - | - |  | 1.31(0.72-2.39) | .37 | 1.37(0.72-2.60) | .33 |
|  | 3 or above | 0.52(0.19-1.41) | .20 |  | - | - |  | 0.52(0.19-1.41) | .20 | 0.50(0.18-1.40) | .19 |
| Number of casual man sexual partners (with no money transaction) in the past six months | | | | | | |  |  |  |  |  |
|  | 0 | 1.00 | - |  | - | - |  | 1.00 | - | 1.00 | - |
|  | 1 | 0.91(0.55-1.57) | .73 |  | - | - |  | 0.91(0.55-1.52) | .73 | 0.93(0.55-1.57) | .78 |
|  | 2 | 1.35(0.76-2.38) | .30 |  | - | - |  | 1.35(0.76-2.38) | .30 | 1.10(0.60-2.02) | .75 |
|  | 3 or above | 1.03(0.51-2.09) | .93 |  | - | - |  | 1.03(0.51-2.08) | .93 | 1.13(0.54-2.35) | .75 |
| Have sex with male sex workers in the past six months | | | |  |  |  |  |  |  |  |  |
|  | No | 1.00 | - |  | - | - |  | 1.00 | - | 1.00 | - |
|  | Yes | 1.55(0.69-3.50) | .29 |  | - | - |  | 1.55(0.69-3.49) | .29 | 1.60(0.67-3.80) | .29 |
| Have sex with women in the past six months | | |  |  |  |  |  |  |  |  |  |
|  | No | 1.00 | - |  | - | - |  | 1.00 | - | 1.00 | - |
|  | Yes | 0.69(0.38-1.28) | .24 |  | - | - |  | 0.69(0.38-1.28) | .24 | 0.85(0.45-1.60) | .61 |
| Drug use for promoting sex in the past six months | | | |  |  |  |  |  |  |  |  |
|  | No | 1.00 | - |  | - | - |  | 1.00 | - | 1.00 | - |
|  | Yes | 1.29(0.83-2.01) | .26 |  | - | - |  | 1.29(0.82-2.01) | .27 | 1.18(0.73-1.89) | .49 |
| **STDs** | |  |  |  |  |  |  |  |  |  |  |
| Have other STDs | |  |  |  |  |  |  |  |  |  |  |
|  | No | - | - |  | 1.00 | - |  | 1.00 | - | 1.00 | - |
|  | Yes | - | - |  | 1.13(0.60-2.09) | .71 |  | 1.05(0.57-1.93) | .87 | 1.06(0.56-2.01) | .85 |
| **HIVST behaviors** | |  |  |  |  |  |  |  |  |  |  |
| Number of HIVSTs | |  |  |  |  |  |  |  |  |  |  |
|  | 2 times or below | - | - |  | 1.00 | - |  | - | - | 1.00 | - |
|  | 3 times | - | - |  | 3.45(2.08-5.74) | <.001 |  | - | - | 3.22(1.92-5.41) | <.001 |
|  | 4 times or above | - | - |  | 7.20(4.64-11.13) | <.001 |  | - | - | 7.23(4.61-11.33) | <.001 |

AOR: adjusted odds ratio; STD: sexually transmitted disease; HIVST: HIV self-testing.

*****Demographic and social characteristics, sexual behaviors were adjusted in the multivariate logistic model.

******Demographic and social characteristics, STDs, and HIVST behaviors were adjusted in the multivariate logistic model.

*******Demographic and social characteristics, sexual behaviors, and STDs were adjusted in the multivariate logistic model.

********Demographic and social characteristics, sexual behaviors, STDs and HIVST behaviors were adjusted in the multivariate logistic model.
