# Supplementary figures and images for: Adherence of HIV Self-Testing Among Men Who Have Sex With Men in China: Longitudinal Study
Source: J Med Internet Res. 2020 Sep 17;22(9):e19627. doi: 10.2196/19627 (PMC7530689; doi:10.2196/19627)

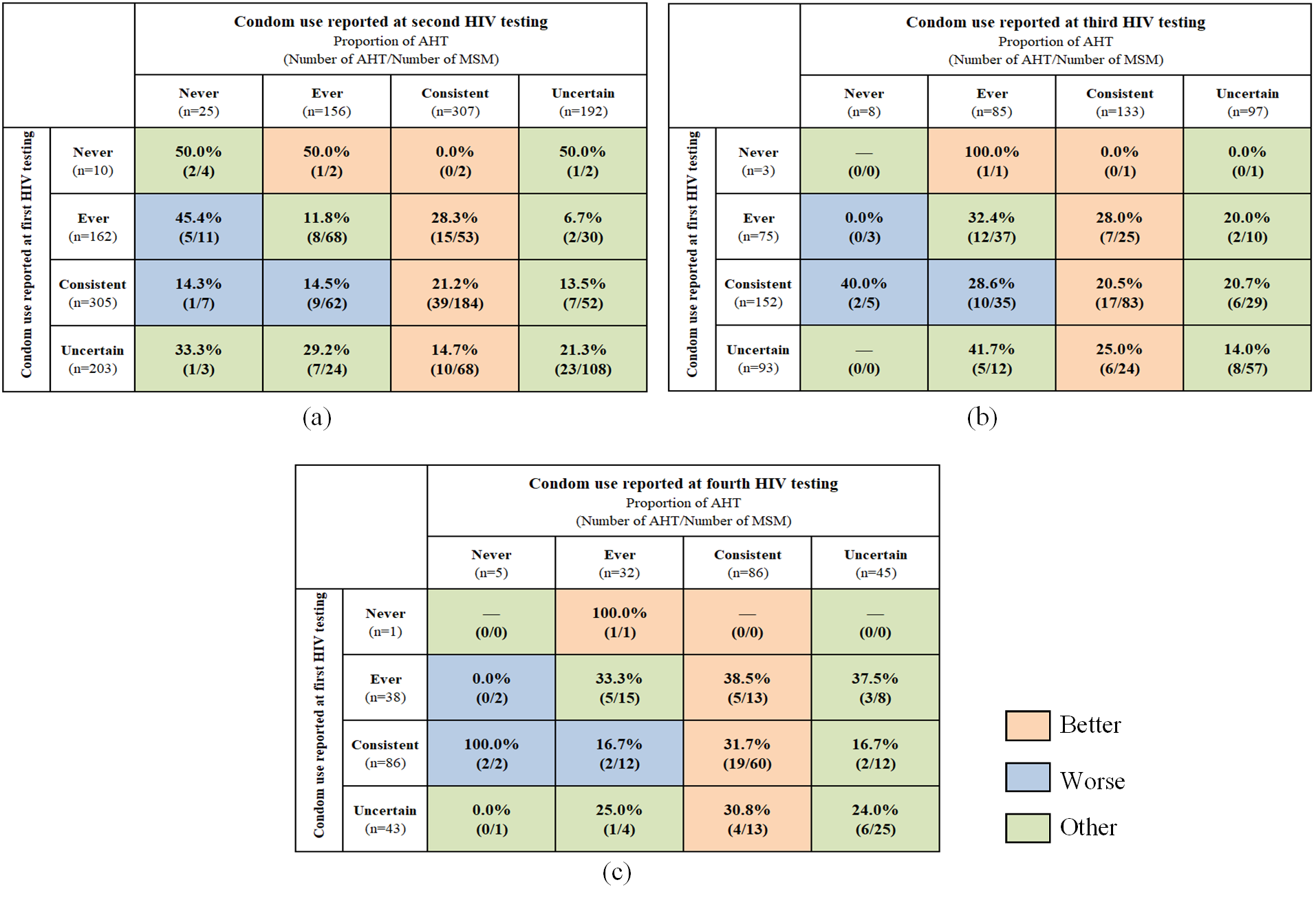

Supplement: Multimedia Appendix 2 [file jmir_v22i9e19627_app2.png]
